# Clinical profile and molecular genetic analysis of alport syndrome in children: a single center experience

**DOI:** 10.3389/fped.2024.1487927

**Published:** 2024-12-23

**Authors:** Aqsa Ahmad, Liang Lijun, Zhang Yan, Ma Yan, Zhao Shuai, Du Wangnan

**Affiliations:** ^1^ The First Clinical Medical College of Ningxia Medical University, Yinchuan, China; ^2^ Department of Pediatrics, General Hospital of Ningxia Medical University, Yinchuan, China; ^3^ Department of Pediatrics, Graduate School, Dalian Medical University, Liaoning, China

**Keywords:** alport syndrome, clinical features, genetic testing, hematuria, children

## Abstract

**Background:**

Alport syndrome (AS) is a multifaceted condition that primarily affects the basement membranes of the kidneys, ears, and eyes. AS is considered the second most common cause of hereditary renal failure, exhibiting varied clinical manifestations across different lifespans. The aim of this study is to investigate the clinical features and genetic profile of AS and to elucidate the genotype-phenotype correlation of AS.

**Method:**

The clinical and genetic data of ten children with AS treated at the General Hospital of Ningxia Medical University between January 2021 and May 2024 were retrospectively analyzed.

**Results:**

Ten children with AS, six male and four female patients, with a mean age of 9 years (ranging from 3 to 15 years) were reported. Hematuria was observed in all individuals, with six cases exhibiting microscopic hematuria and four cases exhibiting macroscopic hematuria. Furthermore, extra-renal manifestations were noted in five cases, encompassing ocular abnormalities (*n* = 2) and hearing impairment (*n* = 3). In total, eight cases displayed mutations in *COL4A5* indicating XLAS, while two cases manifested mutations in *COL4A4* indicating ADAS. Nine different variants were detected, with 3 mutations identified as novel. Two cases underwent histopathological analysis, revealing a thin basement membrane and mild to moderate mesangial proliferation. Three cases were lost to follow-up, while the remaining seven maintained regular visits to our hospital. As of August 1st, 2024, the median follow-up time was 30 (range 24–36) months, and the renal function of the children under observation remained within normal parameters.

**Conclusion:**

In this study, the most commonly observed mutation was glycine substitution. Additionally, patients exhibiting severe mutations showed an increased vulnerability to complications, including proteinuria, ocular lesions, and hearing impairment. Genetic testing emerged as a critical resource for diagnosing AS. Furthermore, early diagnosis is crucial for implementing an appropriate management plan and assessing the prognosis.

## Introduction

AS is a genetically and phenotypically heterogeneous disease that affects the glomerular basement membrane (GBM), cochlea, and ocular basement membrane. Mutations in the collagen IV genes *COL4A3, COL4A4,* and *COL4A5* lead to this condition (1). XLAS is estimated to affect about one in every 2,300 of the normal population (2). AS accounts for 0.5% of recently diagnosed end-stage renal disease (ESRD) patients in adults (3) and 12.9% in children (4). It is distinguished by hematuria, gradual renal failure, unique ultrastructural abnormalities in the GBM, hearing loss, and ocular abnormalities (5, 6). There are four genetic forms of inheritance for AS: X-linked AS (XLAS), autosomal recessive AS (ARAS), autosomal dominant AS (ADAS) and digenic AS.

AS is often diagnosed through pathological examination, often using an electron microscope. But, the results of renal biopsy may not be typical for young patients, leading to misdiagnosis. As a result, genetic testing is the most accurate method of determining a diagnosis, as it also illustrates the method of inheritance and, in some cases, the possibility of early-onset kidney failure and extra-renal complications (7). Diagnosis of AS is crucial since effective and inexpensive therapy with renin-angiotensin-aldosterone system (RAAS) inhibition delays renal failure (8, 9).

The early diagnosis of AS poses challenges and often necessitates a kidney biopsy or genetic testing. Despite significant advancements in the research on the genotype and clinical phenotype of AS-related genes, ongoing technological developments continue to unveil novel genotypes. However, there remains a need for enhanced comprehension of the intricate relationship between the genotype and phenotype of AS. This study undertakes a retrospective analysis of the genetic and clinical characteristics of ten pediatric AS patients treated at our hospital in recent years, aiming to elucidate the genotype-phenotype correlations.

## Method

### Diagnostic criteria for AS

According to the Expert Consensus on Diagnosis and Treatment of Alport Syndrome (Chinese version 2023) (10), ①+ ② or ③ or ④ can be diagnosed as AS if any one of the following conditions are met.

①. The main clinical manifestations include persistent glomerular hematuria or hematuria with proteinuria.

②. The histological examination of renal biopsy shows a diffusely thickened basement membrane or has a varied thickness, the lamina densa is torn and stratified, showing a basket-weave pattern under an electronic microscope.

③. Immunofluorescence examination of type IV collagen *α* chain shows abnormal immunofluorescence staining at *α*3, *α*4, and *α*5 chains or abnormal staining of *α*5 chain in the skin during skin biopsy.

④. Positive genetic test showing a mutation in *COL4A3-5* gene.

This criteria ensured that all patients diagnosed with AS in this study satisfied the above-mentioned conditions.

### Clinical analysis

Individuals <18 years of age with AS were retrospectively analyzed at the General Hospital of Ningxia Medical University from January 2021 to May 2024. The clinical data, encompassing general information such as gender, age of onset, personal history, birth history, and family history, was gathered from the medical records. Additionally, clinical manifestations, including initial symptoms, special signs, extra renal symptoms, and relevant tests such as blood routine, urinalysis, and renal function tests, were compiled.

### Mutation analysis

Through Berry Genomics, comprehensive whole-exome sequencing technology (WES) analysis were conducted for genetic evaluation. Venous whole blood samples (2–3 ml) were collected from the child and parents using EDTA anticoagulant tubes as per established protocols. Genomic DNA extraction was performed using a Blood Genome Column Medium Extraction Kit on the provided DNA. Subsequent to the qualitative examination of the samples, library preparation, sequencing, and bioinformatic analysis were executed. The interpretation of genetic mutations adhered to the American College of Medical Genetics and Genomics (ACMG) guidelines (11). In accordance with these guidelines, pathogenicity was categorized into the following classes: pathogenic (P), likely pathogenic (LP), and variants of unknown significance (VUS). The detection of *COL4A5* mutations was achieved via the MPLA technique.

### Ethical consideration

This study was reviewed and approved by the Institutional Review Board of the General Hospital of Ningxia Medical University. The parents/guardians of the study subjects have given informed consent to this study and signed a written informed consent.

## Results

### Clinical features

**Figure 1:**
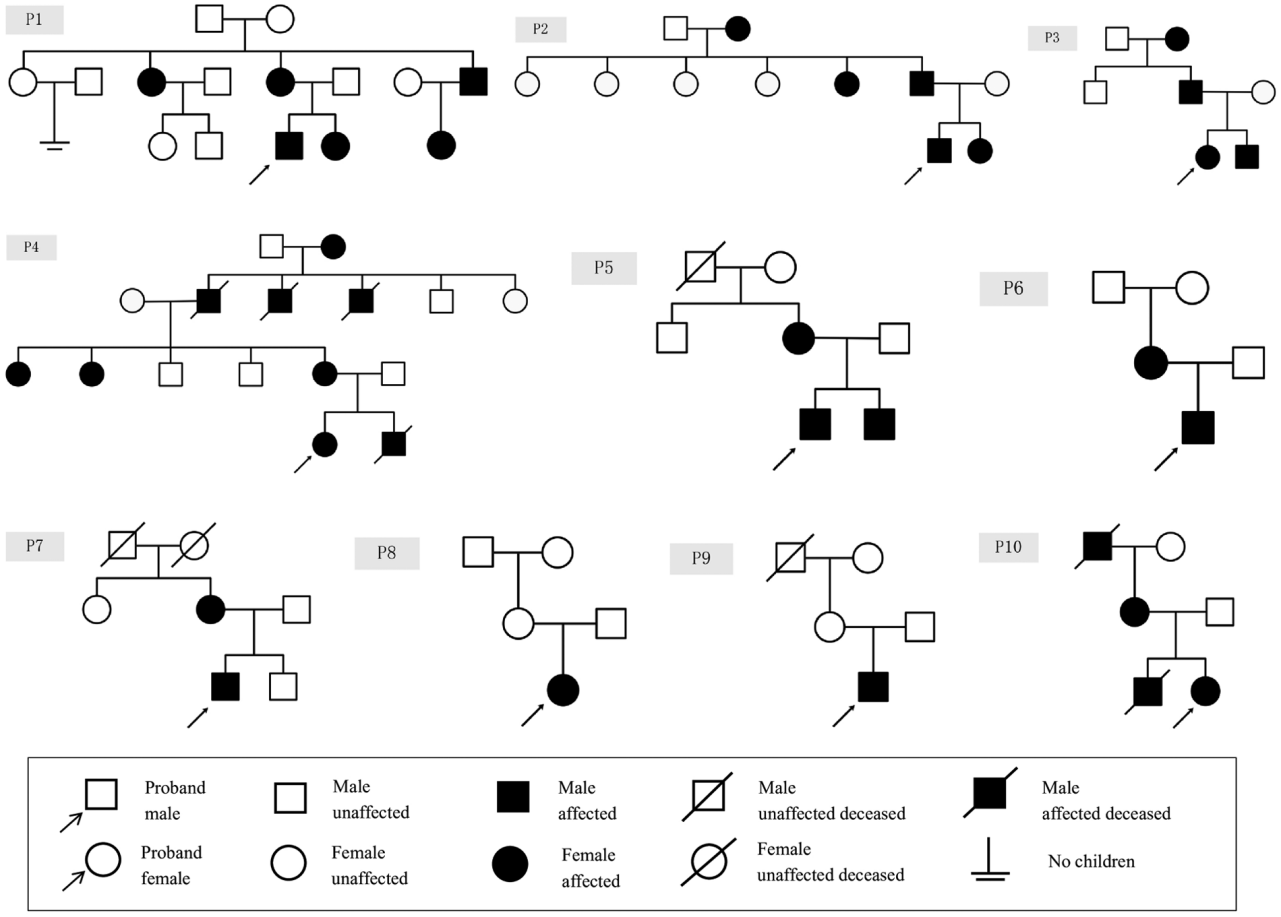
Family tree of children with AS.

The clinical manifestations of ten patients with genetically confirmed AS are detailed in Table 1, while the family tree of the patients is illustrated in Figure 1. The study encompassed six male and four female patients, with a mean age of 9 years (ranging from 3 to 15 years). The laboratory results and auxiliary findings of the participants are outlined in Table 2. Hematuria was observed in all individuals, with six cases exhibiting microscopic hematuria and four cases exhibiting macroscopic hematuria. Additionally, proteinuria was identified in six cases, with three cases demonstrating trace protein levels in urine. Notably, all cases maintained normal renal function throughout the study. Furthermore, extra-renal manifestations were noted in five cases, encompassing ocular abnormalities (*n* = 2) and hearing impairment (*n* = 3). P1, diagnosed with strabismus at age 4 and treated with corrective eyewear, presented with decreased visual acuity but showed no other significant ocular abnormalities upon examination. P2 exhibited a shallow anterior chamber in both eyes on slit-lamp examination, although intraocular pressure (IOP) remained normal at 11 mmHg bilaterally. The cornea was clear, and lens shape and transparency were preserved bilaterally. Fundus examination of both eyes revealed a normal cup-to-disc (C/D) ratio of 0.3 and an artery-to-vein (A/V) ratio of 2:3, suggesting no evidence of glaucomatous damage. Considering the progressive nature of AS, a regular follow-up of 6 months was recommended for both patients. A family history of renal disorders was reported in eight cases, among which P6's mother had been diagnosed with stage 5 chronic kidney disease and had received peritoneal dialysis eight years prior. Renal ultrasound identified anomalies in three cases, indicating enhanced parenchymal echogenicity and unclear delimitation of the cortex and medulla. Only two cases (P7 and P10) underwent renal biopsy, revealing diffuse mesangial proliferation on a light microscope and a thin basement membrane on an electron microscope. P7 also revealed abnormal *α*5-staining upon immunofluorescence staining.

**Table 1:** Clinical features of the children.

Patient ID	Age	Sex	Initial renal manifestation	Family history	Extra renal symptoms	
					Hearing loss	Visual abnormalities
P1	12 y	M	Proteinuria and hematuria	Yes	Normal	Yes
P2	12 y	M	Proteinuria and hematuria	Yes	Normal	Yes
P3	11 y	F	Gross Hematuria	Yes	Not screened	Normal
P4	9 y	F	Gross hematuria	Yes	Not screened	Normal
P5	15 y	M	Proteinuria and hematuria	Yes	Not screened	Not screened
P6	9 y	M	Proteinuria and hematuria	Yes	Binaural sensorineural hearing loss	Normal
P7	13 y	M	Proteinuria and hematuria	Yes	Not screened	Not screened
P8	5 y	F	Gross hematuria	No	Not screened	Not screened
P9	13 y	M	Proteinuria and hematuria	No	Sensorineural hearing loss	Normal
P10	3 y	F	Gross Hematuria	Yes	Sensorineural hearing Loss	Normal

**Table 2:** Laboratory, pathological and genetic features of the children.

Patient ID	Age	Sex	Urinalysis				Ultrasound	Renal Biopsy			Gene	Exon/Intron	Nucleotide change	Amino acid Change	Variant type	Zygosity	Pathogenic analysis	Source of Mutation	Inheritance Mode	Novelty
			Hematuria	Proteinuria	Serum Creatinine	eGFR														
								LM	EM	IF										
					(41–73 umol/L)	(ml/min/1.73 m^2^)														
P1	12 y	M	3+	2+	45.9	121.71	Normal	Not done	Not done	Not done	COL4A5	Exon20	NM_00495.5 c.1276G > C	p.G426R	Nonsynonymous SNV	Hemizygous	Likely Pathogenic (PP1_Strong + PM2 + PP3)	Mother	XLAS	Reported
P2	12 y	M	2+	2+	22	257.25	Normal	Not done	Not done	Not done	COL4A4	Intron3	NM_000092.5 c.114 + 1G > A	–	Splicing	Heterozygous	Pathogenic (PVS1 + PM2 + PP4)	Father	ADAS	Reported
P3	11 y	F	2+	Trace	28	189.12	Normal	Not done	Not done	Not done	COL4A4	Intron34	NM_000092.5 c.3215-2A > G	–	Splicing	Heterozygous	Pathogenic (PVS1 + PM2 + PP1)	Father	ADAS	Reported
P4	9 y	F	3+	Trace	34	145	Diffuse bilateral renal lesion	Not done	Not done	Not done	COL4A5	Exon7	NM_033380.2 c.402A > G	p.Pro134Pro	Synonymous	Heterozygous	Benign	Mother	XLAS	Novel
P5	15 y	M	3+	3+	37	170.54	Normal	Not done	Not done	Not done	COL4A5	Exon41	NM_03338° c.3790G > A	p.G1264S	Missense	Hemizygous	Likely Pathogenic	Mother	XLAS	Novel
P6	9 y	M	3+	3+	24.4	200.43	Diffuse bilateral renal lesion	Not done	Not done	Not done	COL4A5	Exon48	NM_000495.4 c.4613G > A	p.(Trp1538*)	Nonsense	Hemizygous	Pathogenic	Mother	XLAS	Reported
P7	13 y	M	3+	3+	34	171.79	Diffuse bilateral renal lesion	Diffuse mesangial proliferation	TBM	Not available	COL4A5	-	NM_033380.2 c.1216G > T	p.Gly406Cys	Missense	Hemizygous	Likely pathogenic (PM1 + PM5_Supporting + PP3 + PP4)	Mother	XLAS	Novel
P8	5 y	F	3+	Trace	30.4	131.15	Normal	Not done	Not done	Not done	COL4A5	Exon31	NM_000495.5 c.2633G > T	p.G878V	Nonsynonymous SNV	Heterozygous	VUS (PM2 + PP3)	unknown	XLAS	Reported
P9	13 y	M	3+	2+	29.6	204.38	Normal	Not done	Not done	Not done	COL4A5	Exon30	NM0000459.5	–	Deletion	Heterozygous	Unknown	unknown	XLAS	Reported
P10	3 y	F	3+	NIL	17	204	Normal	Diffuse mesangial proliferation	TBM	*α*5-staining positive	COL4A5	-	c.3940C > T	p.Trp.P1314*	Nonsense	Heterozygous	Pathogenic	Mother	XLAS	Reported

LM, light microscope; EM, electron microscope; IF, immunofluorescence staining; TBM, thin basement membrane.

### Genetic features

**Figure 2:**
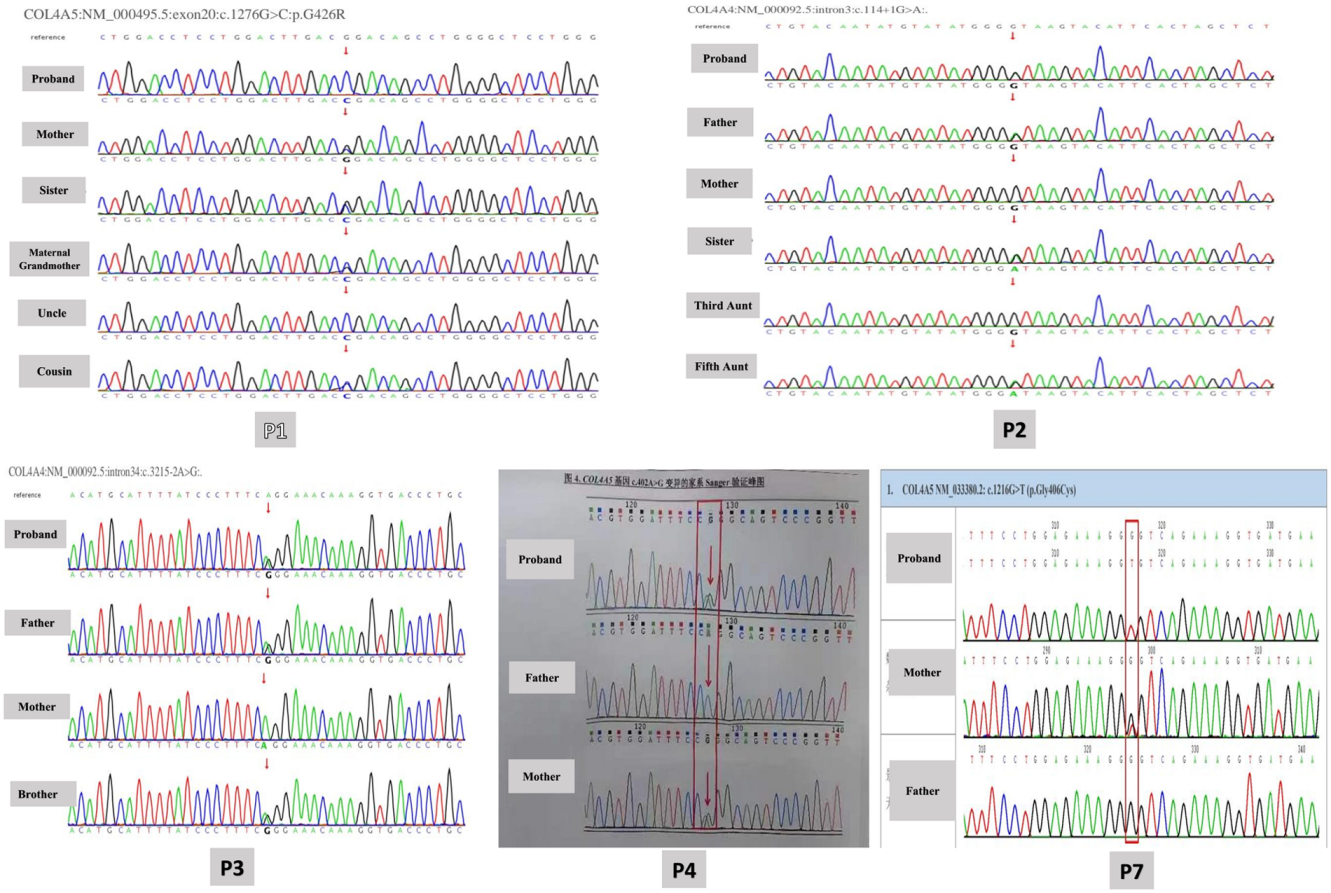
Variants confirmed by Sanger sequencing.

Whole exome sequencing was performed to detect probable disease-causing mutations in each family. After filtering against several databases, including the 1,000 Genome Project, Shenzhou Genome Database, Genome Aggregation Database (gnomAD), and Exome Aggregation Consortium (ExAC), patients' mutations were successfully identified in each family. The detected mutations are shown in Table 2, out of which 5 variants were confirmed by Sanger sequencing as shown in Figure 2. Among the cohort of ten children diagnosed with AS, eight cases displayed mutations in *COL4A5* indicating XLAS, while two cases manifested mutations in *COL4A4* exhibiting ADAS. In total, 9 different variants were identified, including 2 nonsynonymous variants (SNV), 2 splicing variants, 1 synonymous variant, 2 nonsense variants, and 2 missense variants. In addition to these variants, a heterozygous deletion of the *COL4A5* gene located on exon 30 was identified in P9. Notably, seven mutations had been previously documented as the causative mutations for AS in prior studies, with the remaining three mutations recognized as novel findings in this study. Glycine substitution was the most prevalent form of mutation (*n* = 6).

In total, eight cases had X-linked inheritance (six inherited from the mother and two had unknown origin), and two cases had autosomal dominant inheritance (inherited from the father). All the patients had hematuria at diagnosis with stable eGFR. Sensorineural hearing loss was noted in three of the XLAS patients with maternal inheritance, whereas the ADAS patients did not exhibit hearing loss. In the X-linked cohort, severe renal symptoms were observed in several affected relatives on the maternal side, suggesting a higher familial clustering of severe renal involvement. Affected individuals in this cohort, such as P1, exhibited renal manifestations in multiple maternal family members, including his mother, maternal grandmother, aunt, and uncle. Specifically, while P1's maternal uncle developed renal failure and required dialysis, his mother and maternal aunt both presented with microscopic hematuria. Similarly, P4's maternal relatives, including his mother and maternal aunts, also displayed microscopic hematuria. Furthermore, P5's mother had been diagnosed with AS years prior to his own diagnosis. Notably, P6's mother had been diagnosed with stage 5 chronic kidney disease and had undergone peritoneal dialysis eight years ago. Additionally, P7's mother and maternal grandmother also displayed microscopic hematuria. This was notably absent in the autosomal dominant cohort, where family members did not exhibit similar renal manifestations. Specifically, whereas P2's father exhibited no renal symptoms, P3's father presented only with mild microscopic hematuria (2+), highlighting a lack of significant renal involvement in this group.

### Follow-up

Subsequent management of AS patients was tailored to their individual clinical presentations, with the majority (70%) receiving renin-angiotensin-aldosterone system (RAAS) inhibitors, while a portion (30%) received traditional Chinese medicine (TCM). The most commonly used TCM for the treatment of hematuria is Xue'an capsule (血尿胺胶囊), which is made from 4 Chinese herbs: white grass root, small thistle, yellow cypress, and orthosiphon aristatus. It has been reported in various Chinese literature that Xue'an capsule combined with western medicine has achieved commendable results in the treatment of glomerular hematuria (12). Out of the ten children studied, three were lost to follow-up, while the remaining seven maintained regular visits to our hospital, as shown in Table 3. As of August 1st, 2024, the median follow-up time was 30 months (range 24–36), and the renal function of the children under observation remained within normal parameters.

**Table 3:** Follow-up of children with AS.

Patient ID	Treatment (RAASi/TCM)	Baseline eGFR (ml/min/1.73 m^2^)	eGFR at last follow-u (ml/min/1.73 m^2^)	Proteinuria (at diagnosis)	Proteinuria (at last follow-up)	Hematuria (at diagnosis)	Hematuria (at last follow-up)	Follow-up duration (Months)	Lost to follow-up
P1	RAASi	121.71	114.29	2+	1+	3+	3+	30	No
P2	RAASi	257.25	300.57	2+	1+	2+	3+	24	No
P3	RAASi	189.12	Not available	Trace	Not available	2+	Not available	Not available	Yes
P4	TCM	145	146	Trace	1+	3+	3+	26	No
P5	RAASi	170.54	169.24	3+	3+	3+	3+	36	No
P6	RAASi	200.43	148.53	3+	3+	3+	3+	28	No
P7	RAASi	171.79	122.03	3+	3+	3+	3+	30	No
P8	TCM	131.15	Not available	Trace	Not available	3+	Not available	Not available	Yes
P9	RAASi	204.38	204.39	2+	2+	3+	3+	36	No
P10	TCM	204	Not available	NIL	Not available	3+	Not available	Not available	Yes

eGFR, estimated glomerular filtration Rate; RAASi, renin-angiotensin-aldosterone system inhibitors; TCM, traditional chinese medicine.

## Discussion

Arthur C. Alport first identified the disorder in 1927, and it was given the name “Alport syndrome” (AS) in 1961 (13). While AS is considered the most prevalent hereditary kidney disorder. However, there are instances where AS is diagnosed in individuals without any familial history of renal diseases. This underscores the importance of conducting genetic tests in patients exhibiting persistent abnormal urinalysis, regardless of their family history. Notably, here two cases were genetically confirmed to have AS despite the absences of a familial background. The clinical manifestations observed in patients with AS can vary significantly, ranging from isolated hematuria to the onset of renal failure, along with potential extra-renal manifestations, which emphasizes the significance of inter- and intra-familial variability in renal presentations among AS patients (14). Among the families affected by AS, impaired renal function was predominantly noted in patients with mutations in the *COL4A5* gene. In contrast, individuals carrying heterozygous variants of *COL4A4* exhibited more favorable renal outcomes, a finding that aligns with prior studies (15). Hearing loss associated with AS has not been characterized as congenital, and affected individuals typically pass newborn hearing screenings. It is generally identified through audiometric evaluation during late childhood or early adolescence, often manifesting as a bilateral decrease in sensitivity to mid and high frequencies (16–19). Research indicates that the likelihood of experiencing hearing loss before the age of 30 is approximately 60% for patients with missense mutations and can reach up to 90% for those with severe mutations (nonsense mutation, frameshift mutation, mutations at donor or acceptor site, large deletions) related to XLAS (20).

AS is characterized as a renal disorder with rapid progression, emphasizing the need for early diagnosis. A study revealed detection rates for immunofluorescence staining of type IV collagen, renal biopsy via electron microscopy, and genetic testing for AS at 77.8%, 92.6%, and 96.6%, respectively (21). Consequently, kidney biopsy and genetic testing are essential for the accurate diagnosis of this condition. In accordance with the recommendations related to genetic testing and the management of AS, specialists now recommend genetic testing for individuals with persistent hematuria, particularly when there is a suspicion of a heterozygous pathogenic variant in *COL4A3* or *COL4A4* (15). Additionally, cascade testing for first-degree relatives is encouraged due to their increased risk of compromised kidney function. In contrast to conventional gene-panel methodologies, whole exome sequencing (WES) offers enhanced capabilities for identifying potential genetic variants associated with phenotypic manifestations (22). WES also plays a pivotal role in uncovering the modifier genes associated with AS. A recent investigation revealed multiple loci linked to the variations in albuminuria and glomerular filtration rate (GFR) within a mouse model, which includes a locus on the X chromosome connected to X inactivation and another locus on chromosome 2 that encompasses the Fmn1 gene (23). Consequently, performing genetic analyses through WES on patients with AS will provide valuable insights into the intricate relationship between genotypes and phenotypes. It also plays a guiding role for the patient's family members in reproduction.

The current study identifies three variants that have not been previously reported, indicating a wide mutation spectrum of AS. Among the variants identified, two intronic mutations (P2 and P3) occurred at splicing sites, where alterations in the GT-AG box are likely to lead to splicing errors. It can be challenging to differentiate between intronic variants that cause splicing errors and those that are harmless polymorphisms. While various in silico methods exist to evaluate the function of intronic variants, functional analyses are essential to validate the in silico findings (24). Therefore, a thorough investigation of intronic variants is warranted, particularly for those located near conserved splicing sites.

In summary, the identification of these pathogenic variants contributes to a better understanding of the correlation between the phenotypic and genotypic characteristics of AS.

### Limitations

However, this study had some inherent limitations due to its retrospective design. Firstly, the sample size of our study was small and was conducted at a single center, which could have led to biased findings. Therefore, further studies with a larger sample size are highly desirable to validate these findings. The second important limitation is the absence of a clear case explanation of extra renal manifestation, especially that of ocular abnormalities among AS patients. The third limitation of this study is that targeted assessments for ocular findings and hearing impairment were performed in only a subset of patients, therefore, the observed paucity of ocular and hearing abnormalities may not accurately reflect their true prevalence in the cohort. Lastly, lack of renal biopsy data limits our ability to provide direct histopathological correlation with the observed clinical and genetic findings.

## Conclusion

This study presents a cohort of ten Chinese children diagnosed with AS. It is noteworthy that pediatric cases rarely exhibit extra-renal manifestations. The most frequently identified mutation involved glycine substitution. Furthermore, patients with more severe mutations demonstrated a higher susceptibility to complications such as proteinuria, ocular abnormalities, and hearing loss. Genetic testing proved to be an essential tool for the diagnosis of AS. It not only assists in diagnosis but also identifies the location and type of variant, thereby aiding in selecting appropriate treatment plan and predicting prognosis. Early diagnosis and an appropriate management possess significant potential for preventing complications among children.

## Data Availability

The datasets presented in this study can be found in online repositories. The names of the repository/repositories and accession number(s) can be found in the article/Supplementary Material.
